# Optimization and Modeling of Ultrasound‐ and Microwave‐Assisted Extraction of Turmeric to Efficiently Recover Curcumin and Phenolic Antioxidants Followed by Food Enrichment to Enhance Health‐Promoting Effects

**DOI:** 10.1002/fsn3.70093

**Published:** 2025-03-20

**Authors:** Musa Yaman, Sude Nur Arslan, Gözde Gençay, Elifsu Nemli, Müge Yermeydan Peker, Furkan Burak Şen, Esra Capanoglu, Mustafa Bener, Reşat Apak

**Affiliations:** ^1^ Department of Chemistry, Faculty of Science Istanbul University Fatih Istanbul Turkey; ^2^ Department of Food Engineering, Faculty of Chemical and Metallurgical Engineering Istanbul Technical University Maslak Istanbul Turkey; ^3^ Department of Chemistry, Faculty of Engineering Istanbul University‐Cerrahpaşa Avcilar Istanbul Turkey

**Keywords:** bioactive compounds, functional foods, green extraction techniques, turmeric

## Abstract

Phenolic antioxidants and curcuminoids are biologically important molecules playing a crucial role in combating reactive species under oxidative stress conditions. In this study, microwave‐assisted extraction (MAE) and ultrasound‐assisted extraction (UAE) processes for the extraction of phenolic antioxidants and curcumin from turmeric using an ethanol‐water mixture were optimized and modeled with the face‐centered composite design of the response surface methodology. Under optimal conditions, CUPRAC total antioxidant capacity (TAC), curcumin content (CC), DPPH free radical scavenging capacity, ABTS radical scavenging capacity, and total phenolic contents (TPC) of the extracts obtained using MAE, UAE, and automated soxhlet‐assisted extraction were determined to distinguish the water ratio of the ethanolic solvent as the most important operational factor affecting TAC and CC responses. The highest TAC and CC yields were obtained at a 200 μm particle size, 100°C temperature, 30 min time, and 20% water in ethanol conditions for MAE. The highest TAC and CC yields were obtained at a 200 μm particle size, 48 min time, *G* = 600 W ultrasonic power, and 26% water in ethanol conditions for UAE. In addition, the red lentil (R.L.) soup was selected as a model food system and was enriched with extracts obtained by the UAE process. The effects of curcumin addition to a protein‐rich food matrix, spontaneous protein‐curcumin interaction, and the existence of olive oil as an oil/water emulsion delivery system in the prepared soup samples were investigated in association with simulated gastrointestinal digestion. The (R.L + water + 5% turmeric extract) sample was shown to have a higher TPC value than analogous mixtures after in vitro digestion. TPC values of enriched soup samples with olive oil were higher than those enriched without olive oil due to the potential ability of olive oil to provide solubility and stability of curcuminoids together with its potential as a phenolic source. The solubility, oil–water interfacial absorption, and stability of curcuminoids were important in the measured TAC response before and after simulated digestion. Curcumin addition to protein‐rich foods may be recommended considering the health‐promoting effects of functional foods. The proposed extraction processes show a potential to recover bioactive compounds with high efficiency through green chemistry to design new functional foods.

## Graphical abstract

Antioxidants are biologically important molecules playing a crucial role in combating reactive species under oxidative stress conditions. In this study, microwave‐assisted extraction and ultrasound‐assisted extraction (UAE) processes are used for the extraction of antioxidants from turmeric using an ethanol‐water mixture. As a model food system, the red lentil soup was enriched with extracts obtained by the UAE process, and the effects of extract addition to a protein‐rich food matrix and spontaneous protein–curcumin interaction were investigated in association with simulated gastrointestinal digestion.

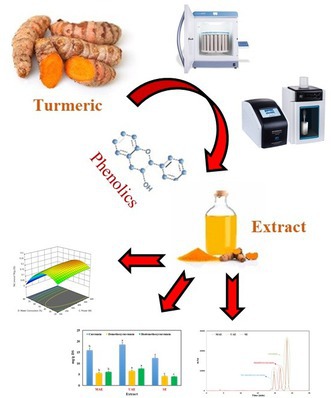

## 1 Introduction

Turmeric is a yellow spice derived from the rhizomes of the *Curcuma longa* L. plant, traditionally utilized in Asian cuisine (Kocaadam and Şanlier 2017). The diverse applications of turmeric involve its utilization in the food industry as a natural coloring agent, flavor enhancer, and spice (Serpa Guerra et al. 2020). In addition to its culinary uses, turmeric finds application in traditional medicine due to its bioactive compounds, of which the primary constituents include curcumin, demethoxycurcumin, and bisdemethoxycurcumin.

Curcumin is recognized as the main active component in turmeric and possesses antioxidant, anti‐inflammatory, anticancer, and antimicrobial properties (Khatun et al. 2021). Thus, curcumin has attracted a great deal of attention in recent years for its pharmacological and physiological activities, leading to its potential for the design of functional foods and dietary supplements (Abd El‐Hack et al. 2021; Jiang et al. 2023a; Racz et al. 2022). However, curcumin exhibits low solubility in water, low absorption, poor stability, and easy degradation within the gastrointestinal tract, rapid elimination from the human body, and low bioaccessibility, resulting in low bioavailability as a major challenge to its use in pharmaceutical and food applications (Racz et al. 2022; Sabet et al. 2021). Recent strategies to overcome these limitations and to increase the bioavailability of curcumin include delivery systems, solid–liquid nanoparticles, solid dispersions, micelles, self‐microemulsions, structural modification, and co‐adjuvants as encapsulation techniques; and nanocomplexation, gelation, electrospraying, and complex coacervation as different methods to improve the solubility and stability of curcumin (Abd El‐Hack et al. 2021; Jiang et al. 2023a; Mirzaee et al. 2019).

Lipophilic nutraceuticals such as curcumin can easily bind to proteins having an amphiphilic nature by hydrophobic interactions through hydrophobic amino acids, and form nano‐complexes (Tang 2020; Zou et al. 2016a, 2016b). The use of plant proteins like barley protein (Jiang et al. 2023b), soy protein isolate (Chen et al. 2015), pea protein (Vijayan et al. 2021), almond, cashew, coconut, and oat milk proteins (Zheng et al. 2021), as well as animal proteins like whey protein (Li et al. 2015; Vijayan et al. 2021), whey protein nanofibrils (Mohammadian et al. 2019), caseinate (Pan et al. 2013), and egg white proteins (Dabbagh Moghaddam et al. 2019) as carriers to protect and deliver curcumin have been investigated, rendering the protein complexation ability of curcumin as most promising strategy for improving its stability, water solubility, bioactivity, and bioaccessibility.

Another essential food component to favorably influence solubility, bioaccessibility, and/or bioavailability of curcumin is oil/fat, making it an important constituent of food and drug model systems. Certain chemical reactions and base‐catalyzed degradation of curcumin occur at a faster rate in aqueous than in oil phase, leading to a reduction in bioavailability through transformation. In such cases, it may be beneficial to maintain curcumin‐like bioactives in an oil phase for an extended period of time throughout the gastrointestinal tract (GIT) (Sabet et al. 2021). For example, the study by Franklyne et al. (2018) emphasized the potential of olive oil nanoemulsions as a delivery vehicle for curcumin, citing their excellent release characteristics and ability to protect curcumin in an aqueous environment. The effect of oil types on the stability and bioaccessibility of curcumin was demonstrated when excipient emulsions were subjected to simulated gastrointestinal conditions, which was attributed to differences in the molecular composition and physicochemical properties of the oils in the study by Zou et al. (2016a, 2016b).

In vitro digestion methods for the investigation of the gastrointestinal behavior of foods and pharmaceuticals, which generally include oral, gastric, and small intestinal phases, and occasionally large intestinal fermentation, attempt to mimic physiological conditions in vivo, taking into account the presence of digestive enzymes and their concentrations, pH, digestion time, and salt concentrations (Minekus et al. 2014). While human nutritional studies are still considered as the “gold standard” for addressing diet‐related questions, in vitro digestion models provide a valuable alternative to animal and human models for the rapid screening of food ingredients due to their flexibility, accuracy, and reproducibility (Minekus et al. 2014; Lucas‐González et al. 2018; Sensoy 2021). It is of paramount importance to be careful when selecting the ingredients, such as curcumin, for the design of new functional foods, model food systems, and/or delivery systems. Furthermore, it is essential to conduct a thorough investigation on the gastrointestinal digestion fate of these ingredients, given the inherent complexity of real food systems.

The process of bioactives extraction from natural sources such as plants is a critical step in isolating these compounds, allowing for their subsequent characterization and utilization in various applications, ranging from pharmaceuticals and nutraceuticals to food and cosmetic industries (Câmara et al. 2021). Analytical separation techniques, including extraction methods such as solvent extraction, supercritical fluid extraction, microwave‐assisted extraction (MAE), and ultrasound‐assisted extraction (UAE) play a pivotal role in efficiently extracting and concentrating these bioactive compounds. These techniques need to be optimized for harnessing the full potential of natural sources and facilitating the development of sustainable and eco‐friendly processes to obtain valuable bioactive components (Contieri et al. 2022; Putra et al. 2023).

MAE, particularly useful for recovering phenolics from natural sources (Shen et al. 2023), involves the application of microwave energy to enhance the extraction process by promoting rapid and efficient heating of the extraction solvent and the sample matrix. The advantages of MAE include reduced extraction times, lowered solvent consumption, and increased extraction yields compared to conventional extraction methods. MAE offers a streamlined and effective approach to isolating phenolics from plant materials. Microwave energy facilitates the disruption of cell walls, leading to enhanced mass transfer and extraction efficiency (Ekezie et al. 2017). The utilization of MAE in the extraction of phenolic compounds underscores its significance in advancing sustainable and high‐throughput methodologies for the isolation of bioactive components, such as antioxidants and curcuminoids from turmeric, with potential applications in pharmaceuticals, nutraceuticals, and other industries. Dandekar and Gaikar (2002) reported a new open‐vessel system MAE technique using organic solvents for the extraction of curcuminoids from turmeric, where the degree of extraction and purity of curcuminoids depend on the selected solvent and the duration of exposure to microwave energy. Bener et al. (2016) optimized the MAE extraction of curcumin from turmeric using methanol and reported the antioxidant and antiradical activities of the obtained extracts. Doldolova et al. (2021) modeled and optimized the MAE process of antioxidants and curcuminoids using natural deep eutectic solvents (NADES).

UAE harnesses the power of ultrasonic waves to enhance the extraction process by inducing cavitation and microstreaming phenomena (Holkar et al. 2019). Ultrasonic waves create microscopic bubbles in the solvent, and upon their collapse, generate localized high temperatures and pressures, facilitating the release of target compounds from the sample matrix (Chemat et al. 2017). UAE can significantly reduce extraction times, increase extraction yields, and improve the overall efficiency of the extraction process compared to traditional methods. The mechanical forces generated by ultrasound assist in disrupting cell structures, aiding in the efficient release of phenolic compounds from plant materials (Vilkhu et al. 2008). Patil et al. (2021) used the UAE technique for the extraction of curcuminoids from *Curcuma longa* L. with different NADES and performed the optimization of different operational parameters, constituting an efficient, cost‐effective, and green alternative to conventional solvent extraction for curcuminoids. Yang et al. (2020) demonstrated that the bioactivities of turmeric extracts obtained by UAE had significantly higher antioxidant and antiproliferative activity than those obtained by traditional extraction techniques.

The success of MAE and UAE methods heavily depends on the optimization of operational factors, influencing the yield, selectivity, and quality of the extracted compounds. In a closed‐vessel MAE system, for instance, microwave temperature and extraction time can dramatically affect the rate of cell wall disruption and solubilization of target compounds, leading to either enhanced recovery or degradation of sensitive molecules (Ferrara et al. 2023). Similarly, in UAE, ultrasonic power and sonication time must be carefully controlled to maximize cavitation effects, which improve mass transfer without causing excessive thermal degradation of the bioactives (Shen et al. 2023). Optimization of these factors can achieve maximum extraction efficiency while preserving the integrity of thermolabile compounds. Failure to optimize these parameters may result in lower yields, degradation of target molecules, or inefficient solvent use, all of which can compromise the environmental and economic advantages of these methods. Response surface methodology (RSM) is often employed for this purpose, providing a systematic and statistically robust approach to assess the interaction effects of multiple variables, thus identifying optimal conditions that ensure the highest extraction efficiency with minimal resource consumption. This approach both maximizes the recovery of bioactive compounds and aligns the extraction process with principles of green chemistry, making the methods more sustainable and scalable for industrial applications (de Souza Mesquita et al. 2024).

In this study, MAE and UAE operational parameters were modeled and optimized by a Face Centred Central Composite Design (FCCD) which comes under the RSM approach using an ethanol‐water mixture for the extraction of curcumin and antioxidants from turmeric. MAE stands out for its accelerated extraction rates, improved yields, multiple sample handling, automation convenience, energy efficiency, and low solvent consumption. UAE offers significant advantages by enhancing the efficiency, improving yield, and reducing extraction time. The amount of curcumin in the extracts was measured using high performance liquid chromatography‐diode array detection (HPLC‐PDA) system, and the antioxidant capacity was determined using the “cupric reducing antioxidant capacity” (CUPRAC) method (Apak et al. 2004). Additionally, an extract was obtained by applying automated Soxhlet‐Assisted Extraction (SAE) and its antioxidant properties were evaluated together with MAE and UAE extracts prepared under optimal conditions. This study represents a unique contribution by optimizing and modeling two innovative and green extraction techniques, MAE and UAE, while distinctly investigating the suitability of the obtained extracts to functional food designs—setting it apart from several similar studies. While there has been a notable increase in the number of in vitro food studies conducted recently, the actual food matrix is significantly more intricate than the models used in these experiments, necessitating the execution of trials utilizing real food systems and/or foods for the purpose of functional food design, with the objective of incorporating bioactive components and investigating the in vitro gastrointestinal fate and the interaction between food components in these designed products. The objective of the food application in this study was to develop a fortified red lentil soup with the addition of curcumin extract, based on the traditional red lentil soup recipe. In this context, fortified red lentil soup samples were prepared with extracts at two levels obtained by the UAE process, with the main basic ingredients. Furthermore, fortified red lentil soup samples were prepared with the incorporation of olive oil, which is another basic ingredient in traditional recipes, as a second sample group. This is due to the fact that the presence of oil in food matrices has been demonstrated to influence the solubility, stability, and bioaccessibility/availability of lipophilic bioactive compounds, particularly during in vitro gastrointestinal digestion, due to the formation of micelles by the activity of digestive enzymes. Another reason for choosing olive oil as the oil phase and source of phenolic compounds in the formulation is to observe the effects of antioxidant phenolics present in olive oil on the total phenolic content and antioxidant activity of the final products having a complex matrix and their ability to protect sensitive compounds such as curcuminoids from environmental, processing, and in vitro gastrointestinal digestion effects, with an additional aim of benefiting from the solubilization potential for lipophilic compounds. Considering this, fortified red lentil soup samples were divided into two groups, with and without olive oil. The effects of curcumin addition and spontaneous protein‐curcumin interaction in the presence/absence of olive oil were investigated before and after in vitro gastrointestinal digestion.

## 2 Materials and Methods

### 2.1 Chemicals and Instrumentations

Chemicals with analytical grades were obtained from different suppliers. Trolox (TR), neocuproine (Nc), 2,2‐diphenyl−1‐picrylhydrazyl (DPPH), the Folin–Ciocalteau reagent, potassium persulfate, acetic acid, curcumin, demethoxycurcumin, bisdemethoxycurcumin, ethanol, and methanol were purchased from Sigma‐Aldrich (St. Louis. Mo., USA). Sodium hydroxide, sodium carbonate, potassium sodium tartrate tetrahydrate, ammonium acetate (NH_4_Ac), copper(II) chloride dihydrate, and copper(II) sulfate were purchased from Merck (Darmstadt, Germany). 2,2′‐Azino‐bis(3‐ethylbenzothiazoline‐6 sulphonic acid) (ABTS) was purchased from Fluka (Buchs, Switzerland). Turmeric root was purchased from the commercial market.

The basic instruments used in the study and their sources were: UV–1900i UV–vis spectrophotometer (Shimadzu, Japan), LC‐2050C HPLC system equipped with PDA‐7000 detector (Shimadzu, Japan) using InertSustain C18 column (5 μm, 4.6 × 250 mm) (GL Sciences, Japan), ETHOS Easy microwave system with IR temperature control system (Milestone, USA), Scientz‐IID Ultrasonic Processor with 6 mm diameter probe (Scientz, China), SER158/3 Solvent AutoExtractor (Velp, Italy), AS 200 Basic analytical sieve shaker with sieves having different pore sizes (Retsch, Germany), BCA 224 analytical balance (Sartorius, Germany), 8011 EB blender (Waring, USA), PURELAB Quest ultra‐pure water system (Elga, Austria).

### 2.2 Sample Preparation

After the turmeric roots were ground using a blender, the ground sample was passed through the analytical sieving system containing sieves with different pore sizes. The sieved samples were separated depending on their size and stored in a 4°C refrigerator to be used in further studies.

### 2.3 MAE of Turmeric

A mass of 0.2 g of ground turmeric sample was placed in a PTFE vessel and soaked with 20 mL solvent. The operational parameters such as particle size, extraction temperature, extraction time, and water ratio in ethanol for the extraction of antioxidant components are 200 μm average size, 95°C, 25 min, and 20% water (v/v). The temperature in the closed vessel was controlled by the IR sensor of the system. The final extracts were first filtered through a Buchner funnel to the flask under vacuum and then passed through 0.45 μm Chromafil PET‐45/25 syringe filters and stored in the refrigerator at 4°C.

### 2.4 UAE of Turmeric

A mass of 0.2 g of ground turmeric sample was placed in a beaker, and the sample was soaked with 20 mL of solvent. The operational parameters such as particle size, extraction temperature, extraction time, and water ratio in ethanol for the extraction of antioxidant components are 200 μm average size, 40 min, 405 W ultrasonic power, and 35% water (v/v). The final extracts were first filtered through a Buchner funnel to the flask under vacuum and then passed through 0.45 μm Chromafil PET‐45/25 syringe filters and stored in the refrigerator at 4°C.

### 2.5 SAE of Turmeric

A mass of 1.0 g of ground turmeric sample was added to the cleaned and dried thimble, and then 100 mL of ethanol was added and placed in the extraction vessels. The vessels were then placed in the SAE auto‐extractor, and the total extraction time was 8 h 15 min (immersion time 8 h, extraction time 10 min, and cooling time 5 min) at the boiling point of ethanol (78°C). The applied time and temperature were determined according to the study conducted by Shirsath et al. (2017), in which the classical Soxhlet process was used. The final extracts were first filtered through a Buchner funnel to the flask under vacuum and then passed through 0.45 μm Chromafil PET‐45/25 syringe filters and stored in the refrigerator at 4°C.

### 2.6 Total Antioxidant Capacity (TAC) Measurement of Extracts

The TAC values of the extracts were determined using the CUPRAC method (Apak et al. 2004), which involves the combination of 1 mL each of CuCl_2_, Nc, and NH_4_Ac solutions. To this mixture, × mL of the sample extract and (1.1‐×) mL of distilled water were added. Following a 30‐min incubation at room temperature, the absorbance values of the solutions were recorded at a wavelength of 450 nm against a reference solution prepared with the same procedure without adding the sample. The TAC was expressed as TR equivalent (mmol TR/g‐dried sample (DS)) based on the standard calibration curve of TR.

### 2.7 Free Radical Scavenging Capacity (FRS) Measurement of Extracts

The FRS of the extract was determined by measuring its ability to scavenge DPPH free radicals (Sánchez‐Moreno et al. 1998). In summary, × mL of extract, (2‐×) mL of methanol, and 2 mL of DPPH (0.2 mM) were added to a tube. After a 30‐min incubation at room temperature, absorbance values were recorded at 515 nm against methanol. Corrected absorbance values (ΔA) were calculated using Equation (1):

(1) $$\Delta A=A_{\text{DPPH}}–A_{S}$$

*A*_DPPH_ was the initial absorbance of DPPH, and *A*_S_ was the absorbance after sample addition to DPPH. FRC was expressed as TR equivalents (mmol TR/g‐DS) based on the standard calibration curve of TR.

### 2.8 ABTS Radical Scavenging Capacity (ARC) Measurement of Extracts

ARC of the extract was determined by measuring its ability to scavenge the chromogenic ABTS radical cation (ABTS^•+^) (Re et al. 1999). ABTS was dissolved in 50 mL of water at a final concentration of 7.0 mM, and persulfate was subsequently introduced to the solution, achieving a final concentration of 2.45 mM. The resultant ABTS^•+^ solution was allowed to mature at room temperature in the dark for 12–16 h. The ABTS^•+^ solution was diluted 1:10 with ethanol to be used in the method. In summary, × mL of sample extract, (4‐×) mL of methanol, and 1 mL of diluted ABTS^•+^ solution were added to the tube. After 6 min of incubation at room temperature, absorbance values were recorded at 734 nm against methanol. Corrected absorbance values (Δ*A*) were calculated using Equation (2):

(2) $$\Delta A=A_{\text{ABTS}}–A_{S}$$

*A*_ABTS_ was the absorbance of ABTS,^•+^ and *A*_S_ was the absorbance after sample addition to ABTS^•+^. ARC was expressed as TR equivalents (mmol TR/g‐DS) based on the standard calibration curve of TR.

### 2.9 Determination of Total Phenolic Content (TPC)

The TPC of the extracts was determined using the Folin‐Ciocalteau method (Singleton et al. 1999). The preparation of the reagents for the Folin‐Ciocalteau method is summarized below. Lowry A solution: 2% Na_2_CO_3_ solution in 0.1 M NaOH. Lowry B solution is 0.5% CuSO_4_ in 1% NaKC_4_H_4_O_6_. Lowry C reagent was freshly prepared by mixing 50 mL of Lowry A solution with 1 mL of Lowry B solution. The Folin‐Ciocalteau reagent was diluted with distilled water at a 1:3 volume ratio before use. The application of the method is summarized below:

In a test tube, × mL of extract, (1‐×) mL of distilled water, and 2.5 mL of Lowry C were added sequentially, and the tubes were allowed to stand at room temperature for 10 min. Subsequently, 0.25 mL of Folin reagent was added to the mixture, and the capped tubes were subjected to incubation at room temperature for 30 min. The absorbance values of the solutions were recorded at 750 nm against the reagent blank. The total phenolic content was expressed in the unit of mmol TR/g‐DS based on the standard curve of TR standard.

### 2.10 Curcumin Content (CC) Measurement of Extract

**FIGURE 1:**
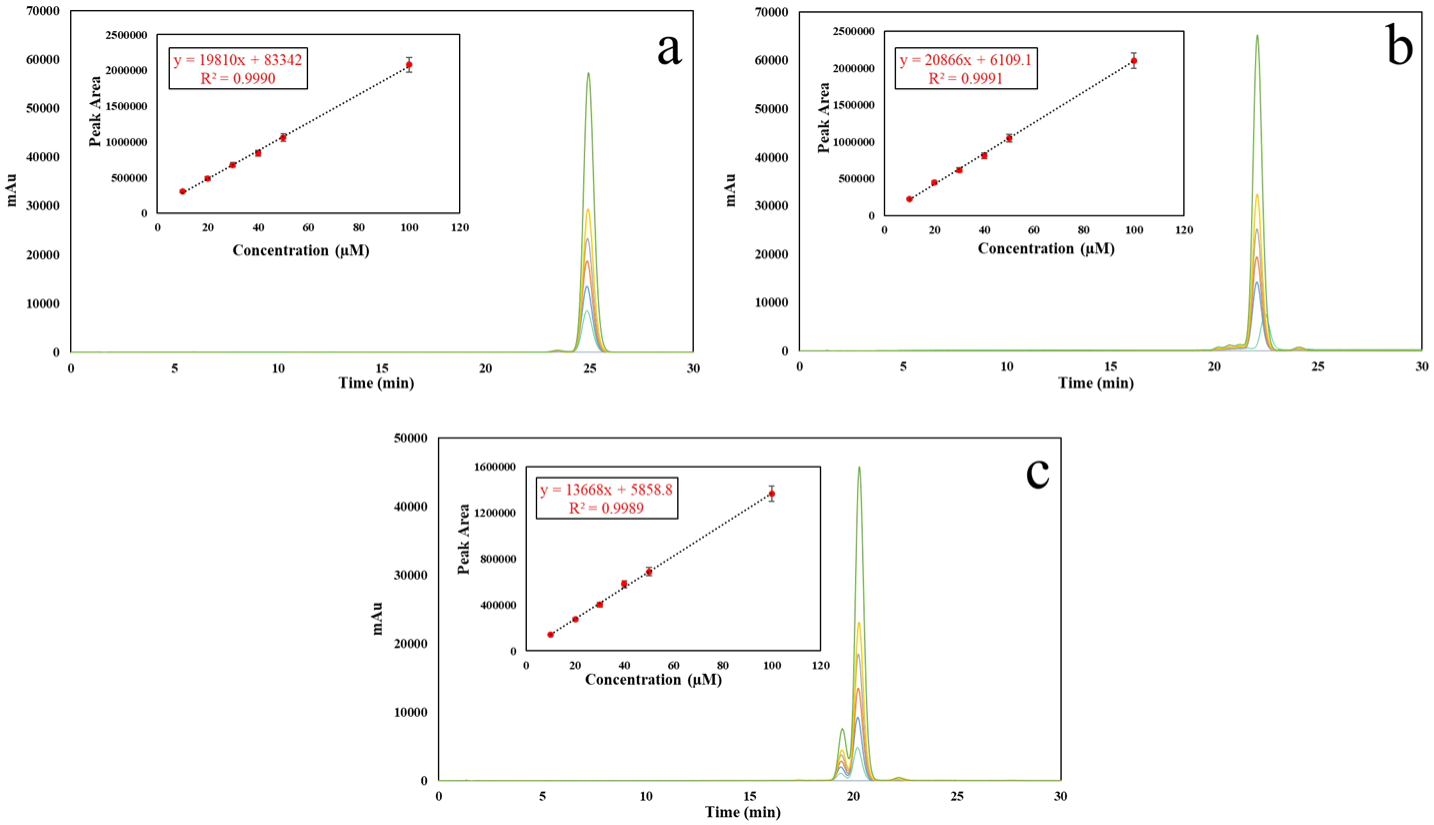
HPLC chromatograms of standard curcuminoids in different concentrations (a: Curcumin, b: Demethoxycurcumin, c: Bisdemethoxycurcumin). Calibration curves of each curcuminoid are given in the inset of the chromatograms.

CC (mg/g‐DS) of the extract was determined using the HPLC‐PDA system described in the study of Wichitnithad et al. (2009) with some minor modifications. An isocratic elution program was used in the reverse‐phase HPLC analysis. The mobile phase consisted of an acetonitrile: acetic acid (2% in double‐distilled water) mixture (40:60, v/v) at a flow rate of 1.6 mL/min. Column temperature, run time, and detection wavelength were used as 33°C, 25 min, and 425 nm, respectively. HPLC chromatograms of standard curcuminoids at different concentrations are summarized in Figure 1. Additionally, the calibration curve and calibration line equation for each curcuminoid are summarized in the chromatograms given in Figure 1. The calibration curves of curcumin, demethoxycurcumin, and bisdemethoxycurcumin were constructed using peak area versus concentration (M) to calculate the CC of the related extract. The limit of detection (LOD) and the limit of quantitation (LOQ) of the curcuminoids were calculated using the equations: LOD = 3.3 (σ/S) and LOQ = 10 (σ/S), respectively, where σ is the standard deviation of the blank‐level response and S is the slope of the corresponding calibration curve. LODs of curcumin, demethoxycurcumin, and bisdemethoxycurcumin were found to be 0.31, 0.36, and 0.45 μM, respectively. LOQs were found to be 1.03, 1.20, and 1.50 μM for curcumin, demethoxycurcumin, and bisdemethoxycurcumin, respectively.

### 2.11 Preparation of Enriched Red Lentil Soup With the Addition of Turmeric Extract

Enriched red lentil soups as a model food system were prepared by adding turmeric extract obtained by the ultrasound‐assisted extraction process at different ratios to observe and understand the effects of turmeric addition to the food matrix, especially rich in protein sources, and protein‐curcumin interactions on the final total phenolic content and antioxidant capacity of enriched soup samples. The red lentils and “Riviera” type olive oil, otherwise referred to as “cooking” type olive oil, which is produced by mixing extra‐virgin olive oil with refined olive oil, were used in the preparation of basic homemade red lentil soups and were purchased from a local market. Firstly, to prepare the basic homemade lentil soup as a control sample, the purchased red lentils and water were mixed at a ratio of 1:9 (by weight) and the obtained mixture was boiled in a cooker for 30 min. After 30 min, the mixture was blended using a household blender. For the preparation of fortified red lentil soups, turmeric extract was added to the soup at a ratio of 5% and 10% (w/w) by reducing the amount of water in the control sample formulation and following the same procedure as for the preparation of the control red lentil soup. Furthermore, the present study was undertaken to gain a more comprehensive insight and to investigate the impact of olive oil on the in vitro gastrointestinal fate of the prepared curcumin‐containing samples with a more complex food matrix and the interaction between the ingredients (such as oils, proteins and curcumin) due to the hydrophobic nature of curcumin, as well as the effects of phenolic compounds of olive oil (especially oleuropein, hydroxytyrosol, and tyrosol) on the final total phenolic content and antioxidant capacity due to its potential as a good phenolic source (Tuck and Hayball 2002; Servili et al. 2009; Tripoli et al. 2005). Enriched red lentil soups were prepared with olive oil added at a ratio of 5% (w/w) by reducing the amount of water as in the case of turmeric extract addition, and with 5% and 10% (w/w) turmeric extract. Also, a further control sample containing red lentils, water, and 5% olive oil was prepared. The codes and formulations of the prepared products are given in Table 1.

**TABLE 1:** Red lentil soup formulations and codes.

Code	Formulation
R.L + W	Red lentils and water (1:9 w/w)
R.L + W + O.O	Red lentils, water and olive oil (5% w/w)
R.L + W + 5% T.E	Red lentils, water and turmeric extract (5% w/w)
R.L + W + 10% T.E	Red lentils, water, and turmeric extract (10% w/w)
R.L + W + O.O + 5% T.E	Red lentils, water, olive oil (5% w/w), and turmeric extract (5% w/w)
R.L + W + O.O + 10% T.E	Red lentils, water, olive oil (5% w/w), and turmeric extract (10% w/w)

### 2.12 Extraction of Polyphenols From Fortified Red Lentil Soup Samples

Solvent extraction of polyphenols in each prepared sample was carried out with a 65% aqueous ethanol solution before the in vitro digestion procedure. Briefly, 5 mL of a 65% aqueous ethanol solution was added to 2.0 g of the sample and then mixed for 15 min in a cooled ultrasonic bath. The obtained mixture was then centrifuged at 4°C and 3420 g for 10 min. After centrifugation, the supernatant was collected, and these extraction steps were repeated a second time for each sample. The supernatants were combined and stored at −20°C until further spectrophotometric analyses.

### 2.13 In Vitro Digestion Procedure

An in vitro gastrointestinal model including the simulation of the mouth, stomach, and small intestine phases according to the standardized method of Minekus et al. (2014) was used to simulate the conditions of food digestion in the human body after ingestion for all test and control red lentil soup samples. The temperatures of all solutions used were adjusted to 37°C before the experiment, and all samples were incubated at this temperature for the duration of the experiment.

#### 2.13.1 Mouth Phase

A mass of 5.0 g of sample was sequentially mixed with 3.5 mL of simulated saliva fluid (SSF), 0.5 mL of α‐amylase stock solution (1,500 U/mL), 25 μL of 0.3 M CaCl_2_ solution, and 975 μL of Milli‐Q water. The final mixture (at pH 7.0) was then incubated in an incubator shaker for 2 min to simulate the mouth phase.

#### 2.13.2 Stomach Phase

A volume of 10 mL of sample from the mouth phase was sequentially mixed with 7.5 mL of simulated gastric fluid (SGF), 1.6 mL of porcine pepsin stock solution (25,000 U/mL) and 5 μL of 0.3 M CaCl_2_ solution, and the pH of the obtained mixture was adjusted to 3.0 by adding 0.2 mL of 1 M HCl solution. The total volume was completed to 20 mL with the addition of distilled water. The final mixture was then incubated in an incubator shaker for 2 h to simulate the stomach phase. At the end of the incubation period, 5 mL of the sample was collected.

#### 2.13.3 Small Intestine Phase

A volume of 15 mL of sample from the stomach phase was sequentially mixed with 8.25 mL of simulated intestinal fluid (SIF), 3.75 mL of pancreatin stock solution (800 U/mL), 1.875 mL of fresh bile solution (160 mM in fresh bile), and 30 μL of 0.3 M CaCl_2_ solution, and the pH of the obtained mixture was adjusted to 7.0 by adding 0.1125 mL of 1 M NaOH solution. The total volume was completed to 30 mL with the addition of distilled water. The final mixture was then incubated in an incubator shaker for 2 h to simulate the small intestine phase. As in the stomach phase, 5 mL of sample was collected at the end of the incubation period.

After all stages of the in vitro gastrointestinal digestion procedure, all samples collected from the stomach and small intestine phases were centrifuged at 4°C and 3420 g for 30 min to remove any large particles. After the centrifugation, all collected supernatants were stored at a temperature of −20°C until further spectrophotometric analyses.

### 2.14 Statistical Analysis

In this study, FCCD (Design‐Expert Software Version 11 (Stat‐Ease Inc., Minneapolis, USA)) was used to determine optimal conditions and investigate the influence of variables. The experimental design, including factors and levels for responses regarding TAC and CC of turmeric extracts in MAE and UAE processes, can be seen in Table 2. Tables 3 and 4 show the effects of the independent variables on the TAC and CC of turmeric extract for MAE and UAE.

**TABLE 2:** Values of the independent factors and their coded forms with their symbols employed in RSM for MAE and UAE optimization.

Factors	Units	Symbol of the variables	Levels		
			−1	0	1
*MAE optimization*					
Particle size (Average)	μm	A	200	490	780
Extraction temperature	°C	B	60	80	100
Extraction time	min	C	5	17.5	30
Water ratio in EtOH	%	D	20	50	80
*UAE optimization*					
Particle size (Average)	μm	E	200	490	780
Extraction time	min	F	10	30	50
Power	W	G	100	350	600
Water ratio in EtOH	%	H	20	50	80

**TABLE 3:** FCCD of the independent factors for the MAE and experimental results of TAC (mmol TR/g‐DS) and CC (mg/g‐DS).

Numbers	The independent factors				TAC	CC
	A	B	C	D		
1	200	60	5	20	0.2101	16.5741
2	780	60	5	20	0.1717	16.3176
3	200	100	5	20	0.2185	15.7807
4	780	100	5	20	0.1839	15.3893
5	200	60	30	20	0.2432	16.3847
6	780	60	30	20	0.2152	16.6567
7	200	100	30	20	0.2710	16.7356
8	780	100	30	20	0.2642	16.6893
9	200	60	5	80	0.1534	7.2697
10	780	60	5	80	0.1023	6.0706
11	200	100	5	80	0.1283	7.7897
12	780	100	5	80	0.1052	6.4737
13	200	60	30	80	0.1156	6.3564
14	780	60	30	80	0.0856	5.3550
15	200	100	30	80	0.1190	6.8588
16	780	100	30	80	0.1175	5.2073
17	200	80	17.5	50	0.2210	11.7453
18	780	80	17.5	50	0.2087	10.4695
19	490	60	17.5	50	0.2106	11.9220
20	490	100	17.5	50	0.2259	10.3932
21	490	80	5	50	0.1998	11.6602
22	490	80	30	50	0.2097	10.6283
23	490	80	17.5	20	0.1994	15.9221
24	490	80	17.5	80	0.0925	6.3547
25	490	80	17.5	50	0.1994	10.7709
26	490	80	17.5	50	0.2136	10.2311
27	490	80	17.5	50	0.2028	10.5373
28	490	80	17.5	50	0.2146	10.6045
29	490	80	17.5	50	0.2116	10.0850
30	490	80	17.5	50	0.2116	10.0202

**TABLE 4:** FCCD of the independent factors for the UAE and experimental results of TAC (mmol TR/g‐DS) and CC (mg/g‐DS).

Numbers	The independent factors				TAC	CC
	E	F	G	H		
1	200	10	100	20	0.2032	16.3227
2	780	10	100	20	0.1289	12.4622
3	200	50	100	20	0.2025	15.6633
4	780	50	100	20	0.1645	13.9525
5	200	10	600	20	0.205	15.2716
6	780	10	600	20	0.1372	12.6417
7	200	50	600	20	0.2451	17.7694
8	780	50	600	20	0.2116	14.7329
9	200	10	100	80	0.0435	3.5544
10	780	10	100	80	0.0381	2.2669
11	200	50	100	80	0.0604	5.1871
12	780	50	100	80	0.0599	4.3987
13	200	10	600	80	0.0525	3.1258
14	780	10	600	80	0.0538	2.5578
15	200	50	600	80	0.0948	6.7181
16	780	50	600	80	0.0847	4.5115
17	200	30	350	50	0.2185	13.6946
18	780	30	350	50	0.2021	10.5661
19	490	10	350	50	0.1952	12.7486
20	490	50	350	50	0.2266	13.0174
21	490	30	100	50	0.1873	10.5092
22	490	30	600	50	0.1844	13.5236
23	490	30	350	20	0.1510	15.7251
24	490	30	350	80	0.0474	3.46296
25	490	30	350	50	0.2116	12.0097
26	490	30	350	50	0.2148	11.8681
27	490	30	350	50	0.2104	11.7246
28	490	30	350	50	0.2266	11.8704
29	490	30	350	50	0.2116	11.0141
30	490	30	350	50	0.1957	11.0145

The experimental plan was chosen randomly to minimize the effects of unexpected changes in response to operational factors. An empirical model was developed that related responses to independent variables using a quadratic polynomial equation. The relationship between independent variables and responses was examined using the analysis of variance (ANOVA) test in the Design‐Expert program.

For spectrophotometric analysis, the mean and standard deviation were calculated from the combination of the results of the analyses performed, and statistical analysis of the data was performed using SPSS software. Differences between treatments were analyzed using ANOVA and Duncan's test at a 95% confidence level (*p* < 0.05).

## 3 Results and Discussion

### 3.1 Modeling and Optimization of MAE

For the models corresponding to TAC and CC responses, *p* < 0.0001 was obtained, indicating a significant relationship between the responses and the independent variables. Water ratio was found to be the most important operational factor affecting TAC and CC responses among all factors. The significance of each coefficient was determined using the F test and *p*‐value summarized in Table 5. Increasing the absolute F value and decreasing the *p*‐value makes the variable significant. It is understood that the models obtained for TAC and CC in the MAE process are statistically significant at the 95% confidence level. According to the ANOVA results, it was found appropriate to use second‐order models to predict TAC and CC. The quadratic model obtained using the independent variables coded for TAC and CC is summarized in Equations (3) and (4).

(3) $$\begin{array}{cc} \mathrm{TAC}= & \;+0.2072-0.0125A+0.0069B+0.0093C-0.0032D\\ & \;+0.0051\mathrm{AB}+0.0051\mathrm{AC}+0.0001\mathrm{AD}+0.0070\mathrm{BC}-0.0052\mathrm{BD}\\ & \;-0.0162\mathrm{CD}+0.0093A^{2}+0.0127B^{2}-0.0008C^{2}-0.0096D^{2} \end{array}$$

(4) $$\begin{array}{cc} \mathrm{CC}= & \;+10.66-0.3814A-0.0883B-0.1363C-4.93D-0.0763\mathrm{AB}\\ & \;+0.0460\mathrm{AD}-0.2966\mathrm{AD}+0.0961\mathrm{BC}+0.1635\mathrm{BD}-0.3894\mathrm{CD}\\ & \;+0.1560A^{2}+0.2063B^{2}+0.1929C^{2}+0.1871D^{2} \end{array}$$

**TABLE 5:** ANOVA for the quadratic equations of FCCD for the TAC and CC for MAE.

	Sum of squares	df	Mean square	*F*	*p*
*TAC response*					
Model	0.0777	14	0.0055	175.52	< 0.0001
A	0.0028	1	0.0028	88.75	< 0.0001
B	0.0009	1	0.0009	27.34	0.0001
C	0.0015	1	0.0015	48.79	< 0.0001
D	0.0509	1	0.0509	1609.43	< 0.0001
AB	0.0004	1	0.0004	13.40	0.0023
AC	0.0004	1	0.0004	13.24	0.0024
AD	1.067 × 10^−7^	1	1.067 × 10^−7^	0.0034	0.9544
BC	0.0008	1	0.0008	25.13	0.0002
BD	0.0004	1	0.0004	13.61	0.0022
CD	0.0042	1	0.0042	133.57	< 0.0001
A^2^	0.0002	1	0.0002	7.04	0.0181
B^2^	0.0004	1	0.0004	13.23	0.0024
C^2^	1.644 × 10^−6^	1	1.644 × 10^−6^	0.0520	0.8227
D^2^	0.0092	1	0.0092	291.48	< 0.0001
Residual	0.0005	15	0.0000		
Lack of Fit	0.0003	10	0.0000	0.7000	0.7054
Pure Error	0.0002	5	0.0000		
Cor Total	0.0782	29			
*CC response*					
Model	448.01	14	32.00	127.82	< 0.0001
A	2.62	1	2.62	10.46	0.0056
B	0.1403	1	0.1403	0.5605	0.4656
C	0.3344	1	0.3344	1.34	0.2658
D	437.23	1	437.23	1746.50	< 0.0001
AB	0.0931	1	0.0931	0.3717	0.5512
AC	0.0338	1	0.0338	0.1352	0.7183
AD	1.41	1	1.41	5.62	0.0315
BC	0.1476	1	0.1476	0.5896	0.4545
BD	0.4277	1	0.4277	1.71	0.2109
CD	2.43	1	2.43	9.69	0.0071
A^2^	0.0631	1	0.0631	0.2520	0.6230
B^2^	0.1102	1	0.1102	0.4404	0.5170
C^2^	0.0964	1	0.0964	0.3850	0.5442
D^2^	0.0907	1	0.0907	0.3621	0.5563
Residual	3.76	15	0.2503		
Lack of Fit	3.29	10	0.3289	3.53	0.0884
Pure Error	0.4665	5	0.0933		
Cor Total	451.76	29			

**FIGURE 2:**
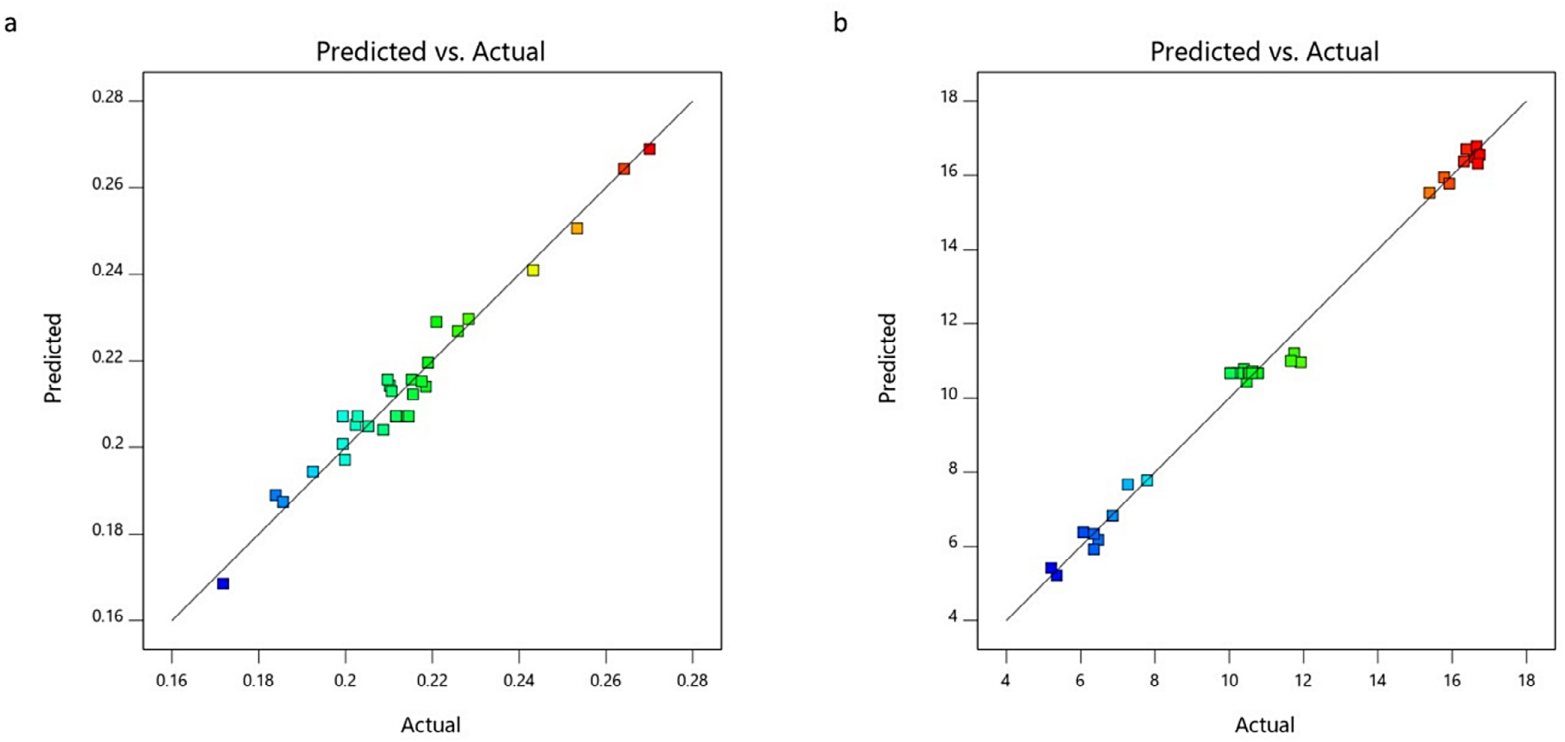
The correlation between the predicted versus actual (a) TAC and (b) CC values of the MAE extract.

The predicted and adjusted *R* ^2^ values were 0.9763 and 0.9883, respectively, for TAC and 0.9661 and 0.9839 for CC. A difference of less than 0.2 indicates that the estimated *R* ^2^ values are consistent with the adjusted *R* ^2^ values. A sufficient sensitivity value measures the signal‐to‐noise ratio, and this value is desired to be greater than 4. The signal‐to‐noise ratio for TAC and CC was found to be 45.655 and 32.663, respectively, and it was decided that the proposed models could be used in the design. In Figure 2, the correlation between the estimated and actual TAC and CC values obtained by the independent variables was examined, and it was concluded that the values predicted from the model were in sufficient agreement with the values obtained from the real data. The highest TAC (0.275 mmol TR/g‐DS) yield was obtained at *A* = 230 μm, *B* = 100°C, *C* = 30 min, and *D* = 29% water conditions. The highest CC (16.78 mg/g‐DS) yield was obtained at *A* = 200 μm, *B* = 86°C, *C* = 30 min, and *D* = 3% water conditions. The association of the highest CC with a much lower water ratio (than that of highest TAC) may be attributed to the low water solubility of curcumin (Rafiee et al. 2019). The highest TAC (0.269 mmol TR/g‐DS) and CC (16.55 mg/g‐DS) yields were obtained at *A* = 200 μm, *B* = 100°C, *C* = 30 min, and *D* = 20% water conditions.

### 3.2 Modeling and Optimization of UAE

*p* < 0.0001 was obtained for the models corresponding to TAC and CC responses, indicating a significant relationship between the responses and the independent variables. Water ratio was found to be the most important operational factor affecting TAC and CC responses among all factors. The significance of each coefficient was determined using the *F* test and *p*‐value summarized in Table 6. Increasing the absolute *F* value and decreasing the *p*‐value makes the variable significant. It is understood that the models obtained for TAC and CC in the MAE process are statistically significant at the 95% confidence level. According to the ANOVA results, it was found appropriate to use second‐order models to predict TAC and CC. The quadratic model obtained using the independent variables coded for TAC and CC is summarized in Equations (5) and (6).

(5) $$\begin{array}{cc} \mathrm{TAC}= & \;+0.2043-0.0136E+0.0163F+0.0100G-0.0619H+0.0040\mathrm{EF}\\ & \;+0.0005\mathrm{EG}+0.0124\mathrm{EH}+0.0071\mathrm{FG}-0.023\mathrm{FH}-0.0010\mathrm{GH}\\ & \;+0.0135E^{2}+0.0141F^{2}-0.0110G^{2}-0.0976H^{2} \end{array}$$

(6) $$\begin{array}{cc} \mathrm{CC}= & \;+11.92-1.07E+0.8333F+0.3631G-5.49H+0.0377\mathrm{EF}\\ & \;-0.0496\mathrm{EG}+0.3992\mathrm{EH}+0.3462\mathrm{FG}+0.2432\mathrm{FH}-0.0318\mathrm{GH}\\ & \;-0.1345E^{2}+0.6182F^{2}-0.2484G^{2}-2.67H^{2} \end{array}$$

**TABLE 6:** ANOVA for the quadratic equations of FCCD for the TAC and CC for UAE.

	Sum of squares	df	Mean square	*F*	*p*
*TAC response*					
Model	0.1368	14	0.0098	60.55	< 0.0001
E	0.0033	1	0.0033	20.61	0.0004
F	0.0048	1	0.0048	29.49	< 0.0001
G	0.0018	1	0.0018	11.25	0.0043
H	0.0689	1	0.0689	427.16	< 0.0001
EF	0.0003	1	0.0003	1.59	0.2264
EG	4.101 × 10^−6^	1	4.101 × 10^−6^	0.0254	0.8755
EH	0.0025	1	0.0025	15.32	0.0014
FG	0.0008	1	0.0008	5.04	0.0402
FH	0.0001	1	0.0001	0.5446	0.4719
GH	0.0000	1	0.0000	0.0979	0.7587
E^2^	0.0005	1	0.0005	2.91	0.1089
F^2^	0.0005	1	0.0005	3.17	0.0953
G^2^	0.0003	1	0.0003	1.94	0.1837
H^2^	0.0247	1	0.0247	153.09	< 0.0001
Residual	0.0024	15	0.0002		
Lack of Fit	0.0019	10	0.0002	1.97	0.2346
Pure Error	0.0005	5	0.0001		
Cor Total	0.1392	29			
*CC response*					
Model	628.76	14	44.91	64.93	< 0.0001
E	20.52	1	20.52	29.66	< 0.0001
F	12.50	1	12.50	18.07	0.0007
G	2.37	1	2.37	3.43	0.0838
H	541.84	1	541.84	783.42	< 0.0001
EF	0.0228	1	0.0228	0.0329	0.8584
EG	0.0394	1	0.0394	0.0569	0.8146
EH	2.55	1	2.55	3.69	0.0741
FG	1.92	1	1.92	2.77	0.1166
FH	0.9460	1	0.9460	1.37	0.2604
GH	0.0162	1	0.0162	0.0234	0.8805
E^2^	0.0469	1	0.0469	0.0678	0.7982
F^2^	0.9900	1	0.9900	1.43	0.2501
G^2^	0.1599	1	0.1599	0.2312	0.6376
H^2^	18.48	1	18.48	26.72	0.0001
Residual	10.37	15	0.6916		
Lack of Fit	9.36	10	0.9362	4.62	0.0525
Pure Error	1.01	5	0.2026		
Cor Total	639.13	29			

**FIGURE 3:**
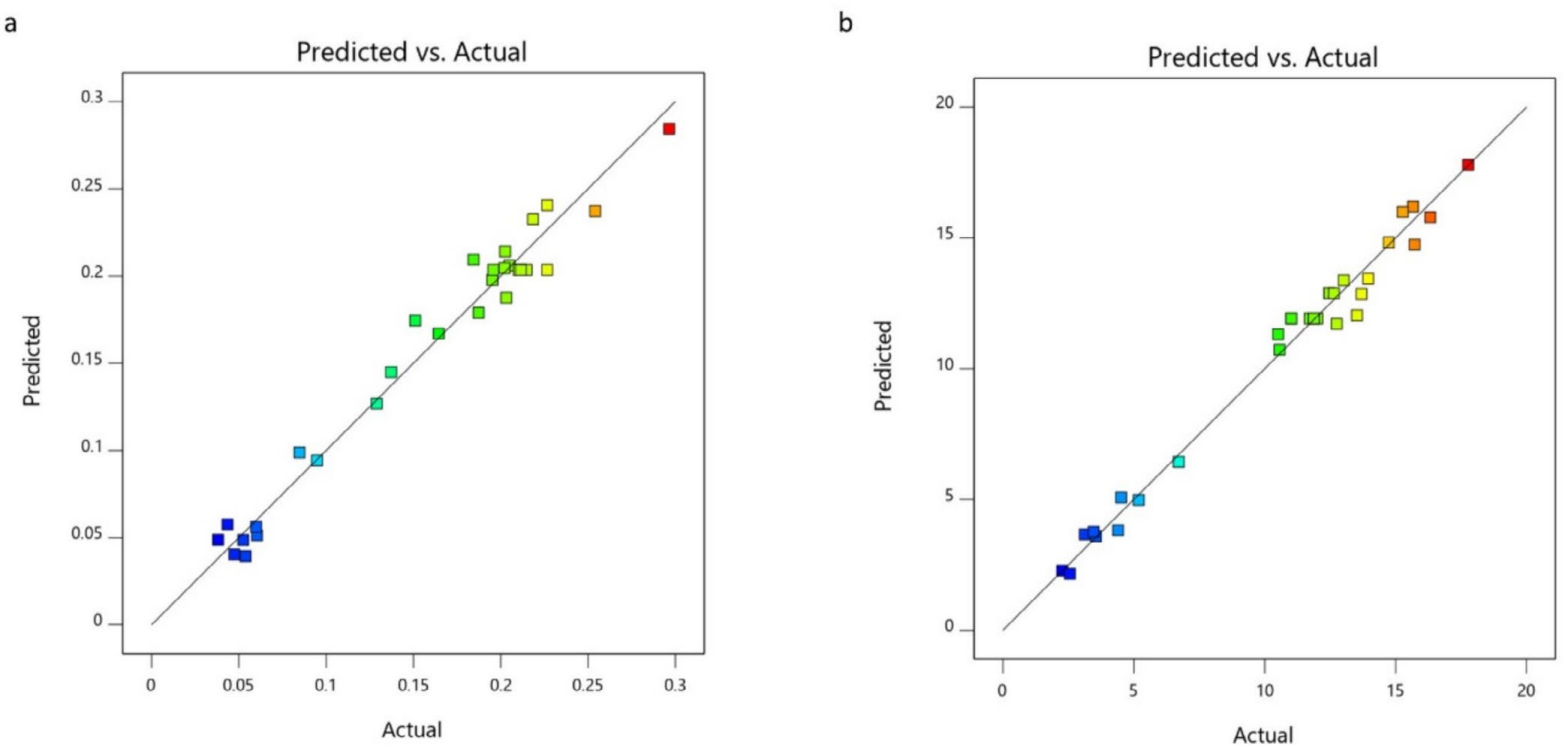
The correlation between the predicted versus actual (a) TAC and (b) CC values of the UAE extract.

The predicted and adjusted *R* ^2^ values were 0.92279 and 0.9664, respectively, for TAC and 0.9243 and 0.9686 for CC. A difference of less than 0.2 indicates that the estimated *R* ^2^ values are consistent with the adjusted *R* ^2^ values. A sufficient sensitivity value measures the signal‐to‐noise ratio, and this value is desired to be greater than 4. The signal‐to‐noise ratio for TAC and CC was found to be 22.662 and 26.579, respectively, and it was decided that the proposed models could be used in the design. In Figure 3, the correlation between the estimated and actual TAC and CC values obtained by the independent variables was examined, and it was concluded that the values predicted from the model were in sufficient agreement with the values obtained from real data. The highest TAC (0.279 mmol TR/g‐DS) yield was obtained at *E* = 251 μm, *F* = 49 min, *G* = 540 W, and *H* = 37% water conditions. The highest CC (17.79 mg/g‐DS) yield was obtained at *E* = 200 μm, *F* = 50 min, *G* = 600 W, and *H* = 20% water conditions. The highest TAC (0.274 mmol TR/g‐DS) and CC (17.40 mg/g‐DS) yields were obtained at *E* = 200 μm, *F* = 48 min, *G* = 600 W, and *H* = 26% water conditions.

### 3.3 Effect of the Extraction Conditions on MAE and UAE

**FIGURE 4:**
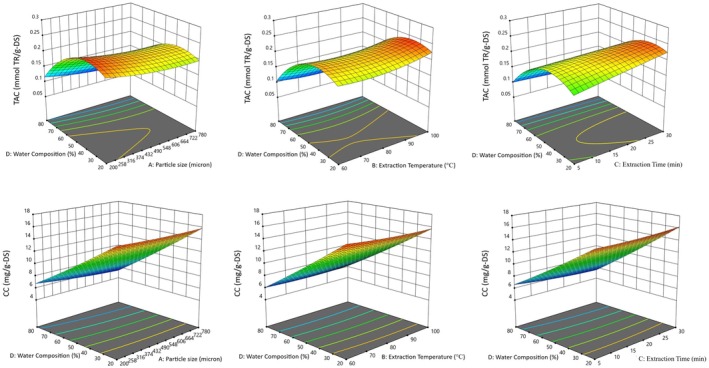
3‐D graphs for the TAC and CC of the MAE extract as a function of operational factors.

**FIGURE 5:**
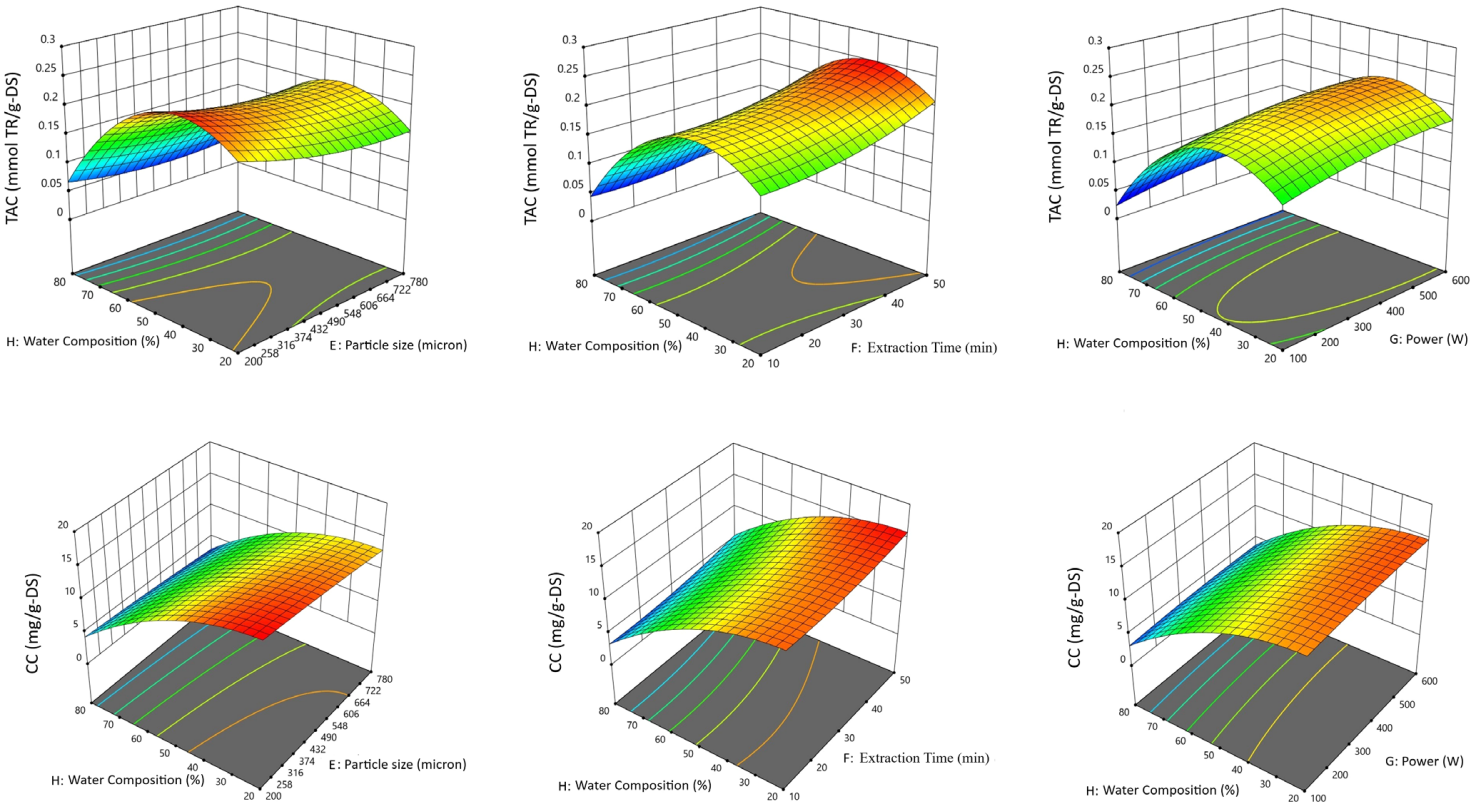
3‐D graphs for the TAC and CC of the UAE extract as a function of operational factors.

The effects of operational factors on MAE and UAE of antioxidants and curcumin from turmeric were investigated. Figures 4 and 5 show the effects of operational parameters on antioxidant and curcumin extraction using MAE and UAE in the form of three‐dimensional (3‐D) graphs. Water composition (%) was found to be the most important parameter in both extraction techniques. In both MAE and UAE processes, when the ethanol ratio increases and the water ratio decreases in the extraction of antioxidants and curcumin, the extraction efficiency increases significantly (Figures 4 and 5). Although curcumin and antioxidants showed similar trends in both MAE and UAE methods with changes in water composition, as the percentage of water reduced, the extraction of curcuminoids increased dramatically, whereas antioxidant extraction reached a maximum at a certain point. In addition to the highly alcohol‐soluble curcuminoids, turmeric also contains small amounts of water‐soluble antioxidants, such as phenolic compounds and turmerin, an antioxidant peptide (Srinivas et al. 1992). As a result, during the extraction process, the decrease in the percentage of water in ethanol leads to a limitation in the increase of total antioxidant capacity (TAC) beyond a certain threshold. The increase in curcuminoid extraction can be explained by the low solubility of the relevant components in water (Rafiee et al. 2019). As a result, curcuminoids, which are the major bioactive components in turmeric, determine the solvent‐based trend in extraction efficiency. Martinez‐Correa et al. (2017) reported the effectiveness of the use of ethanol in the extraction of bioactive antioxidant compounds from turmeric and that the extract had greater antioxidant activity compared to water extract. Solvent type is one of the most important parameters for efficient MAE, and solvent selection should take into account the affinity of the target compound as well as its ability to absorb microwave energy. Ravindran et al. (2009) obtained the highest curcuminoid content and extraction efficiency by MAE using 95% ethanol and reported that the relevant solvent mixture had optimum dielectric constant, viscosity, and solubility for the target compound. Sahne et al. (2016) reported that 95% ethanol exhibited important properties such as sufficiently high polarity, low viscosity, surface tension, absorption of ultrasound energy, and high extraction efficiency compared to other solvents in the UAE process for turmeric extraction. The polarity index of 95% EtOH is not significantly lower than that of 50% EtOH (Rahman et al. 2013). Studies have shown that the polarity of ethanol increases the permeability of the cell wall and thus the extraction efficiency (Yadav et al. 2012). Ethanol solvent has a high capacity to absorb and transmit ultrasound energy during the UAE process and also determines the cavitation intensity based on physicochemical properties (Singh et al. 2022). Examination of the 3‐D diagrams shows that the increase in particle size reduces the efficiency of the MAE and UAE processes for the extraction of both antioxidants and curcumin (Figures 4 and 5). Previous studies have generally reported that reducing the sample particle size has a positive effect on the amount of bioactive compounds obtained. Makanjuola showed that particle size had a positive effect on the antioxidant content of tea and ginger extracts (Makanjuola 2017). Reducing the size of plant substrates before extraction maximizes the surface area, which increases the mass transfer of the active ingredient from the plant material to the solvent (Handa 2008). Wang and Helliwell reported that the contents of some individual antioxidants in ground tea samples were 36% higher than in unground tea samples (Wang and Helliwell 2001). Similarly, Vuong et al. (2011) reported that for green tea, a particle size of 0.25–1 mm resulted in higher catechin extraction than a particle size > 1 mm. Prasedya et al. (2021) studied the effect of sample particle size on the phytochemical composition and antioxidant activity of brown macroalga *Sargassum cristaefolium* . Extraction was performed from dried macroalgae powders with various particle sizes (> 4000 μm, > 250 μm, > 125 μm, > 45 μm and < 45 μm) and it was reported that the extract yield was higher at smaller particle sizes. On the other hand, it has been reported that a critical particle size can be reached such that a further decrease in particle size cannot lead to a further increase or decrease (Makanjuola 2017). It should also be taken into account that very small powder particle sizes may become slimy during extraction and create difficulty during filtration (Makanjuola 2017). Temperature is important for the desorption and dissolution of target compounds using solvents, and since temperature is one of the main factors contributing to extraction efficiency in the MAE process, determining the optimum extraction temperature is critical. Generally speaking, the solubility of components in solvents increases as temperature increases. It is likely that with increasing temperature, the solubility of analytes in the active regions of the sample matrix increases, leading to an increase in extraction efficiency. It has been reported in many studies that extraction efficiency increases as the operating temperature increases, but structural degradation of phenolics with antioxidant properties may occur at high temperatures (Bener et al. 2016; Wettasinghe and Shahidi 1999). Thermal degradation for the phenolic components of the sample is inevitable during the MAE process at high operating temperatures and extreme extraction times, and therefore the maximal temperature is limited to 100°C. Therefore, no thermal deterioration was found in the selected temperature range in our study. As a result, it would be better to avoid very high temperatures, considering the possible degradation of antioxidant compounds as well as economic and safety concerns. It is known that there is a generally positive parallelism between extraction time and extraction efficiency (Lucchesi et al. 2007). During the extraction period, contact and wetting of the solid sample with the solvent, dissolution of the target components by the solvent used for extraction, and release of the solvent from the sample occur, requiring a certain time for these steps (Gan and Latiff 2011). In this study, increments in extraction efficiency were observed during both the MAE and UAE processes (Figures 4 and 5). However, the increase in extraction efficiency was not exactly proportional to extraction time throughout the process. It is known that some bioactive compounds degraded during long extraction times due to thermal exposure in the MAE process and the negative effects of long‐term ultrasonic irradiation in the UAE process (Mendes et al. 2016). Due to these conditions, very prolonged extraction times should be avoided. It was observed that the extraction efficiency of antioxidants and curcumin increased with increasing ultrasonic power in the UAE process (Figures 4 and 5). Increasing ultrasonic power can enhance the opening of cracked or damaged cell walls, which can increase solute diffusion, interfacial turbulence, and local energy dissipation. These physical effects are responsible for the increased extraction at higher ultrasonic power (Gao et al. 2009; Kulkarni et al. 2008). In this study, an increase in extraction efficiency was observed with increasing power input over the entire power range examined, but cases to the contrary have been reported (Balachandran et al. 2006; Lou et al. 2010). When very high ultrasonic power is used, degradation of the extracted compounds may be observed and temperature control may also become difficult. Therefore, it is very important to optimize the ultrasonic power before extraction.

### 3.4 Antioxidant Efficacies and Curcuminoid Content of Turmeric Extracts

**FIGURE 6:**
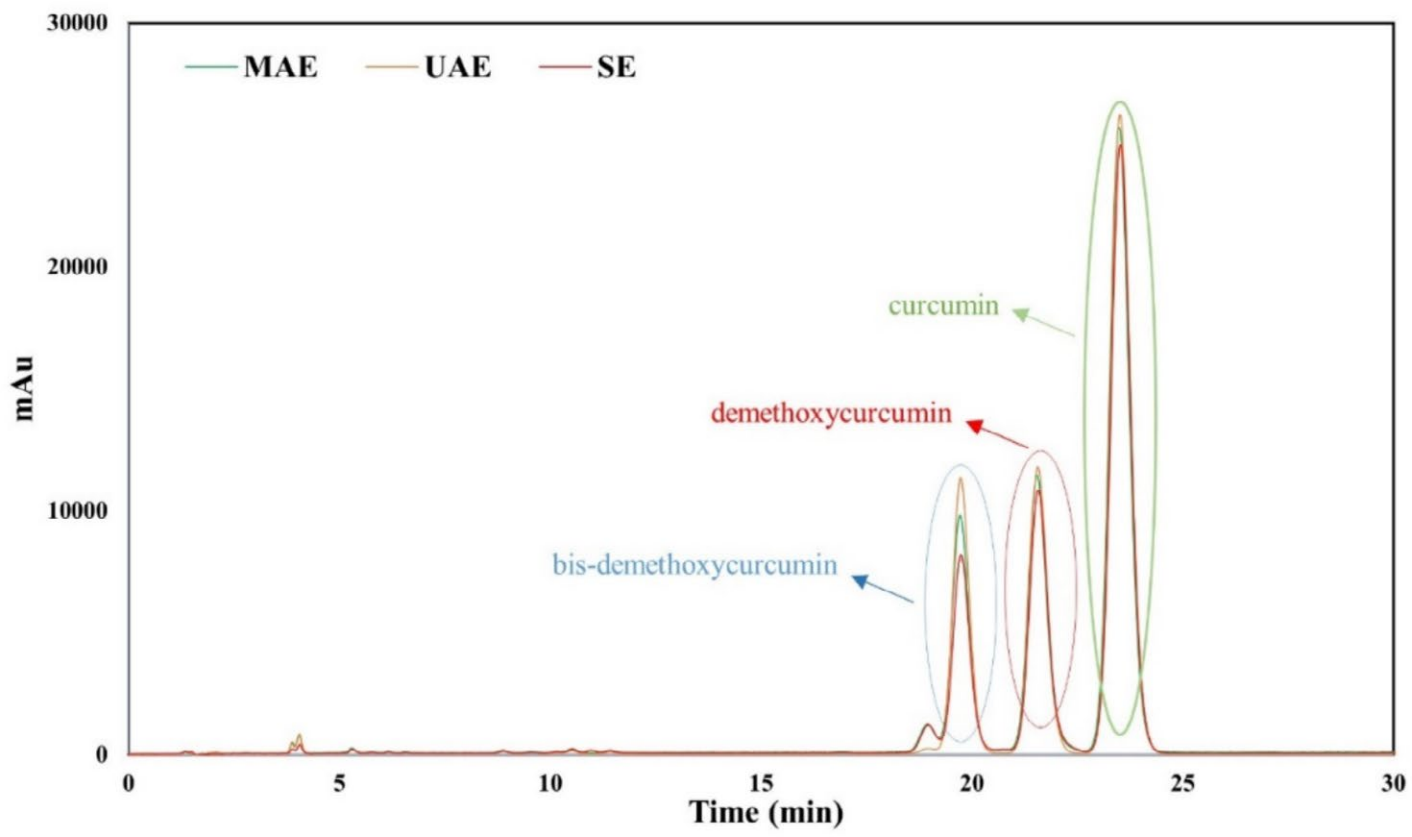
HPLC chromatograms of turmeric extracts obtained by different extraction techniques.

**FIGURE 7:**
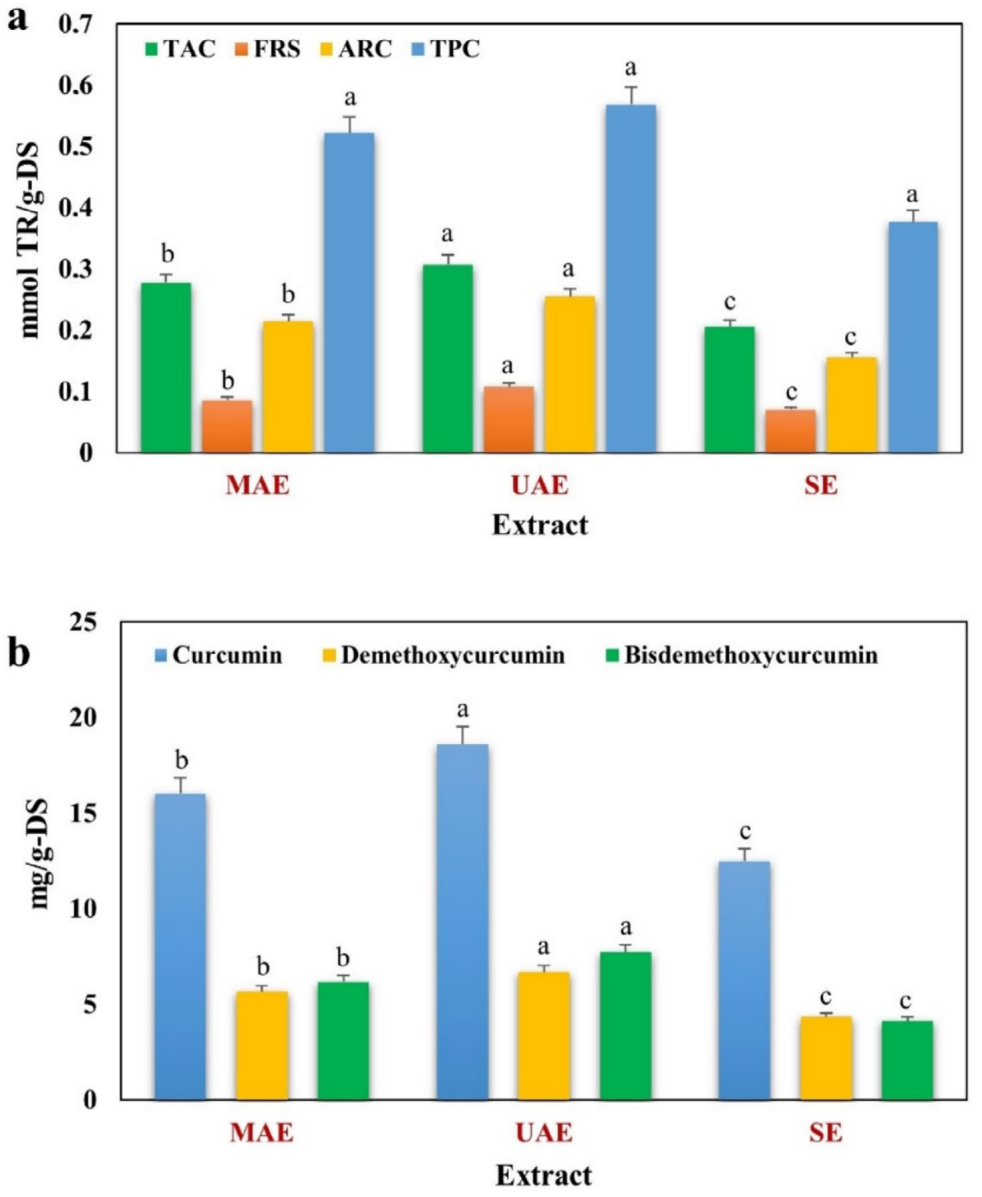
(a) Antioxidant activities and (b) Curcuminoid contents of turmeric extracts obtained by different extraction techniques (Different letters in the columns represent statistically significant differences (*p* < 0.05)).

Turmeric extracts were obtained using MAE and UAE techniques under optimal conditions for the extraction of antioxidants and curcuminoids. The curcuminoids (curcumin, demethoxycurcumin, and bisdemethoxycurcumin) of the obtained extracts were determined using the HPLC‐PDA system, and the resulting chromatograms are presented in Figure 6. TAC, FRS, ARC, and TPC values of the turmeric extracts to evaluate the antioxidant activities were found through spectrophotometric analyses (Figure 7). All the obtained data were compared with the data of extracts using conventional heat‐based automatic SAE. Antioxidant activities and curcuminoid contents obtained with the MAE and UAE systems were significantly higher than those with the SAE system. On the other hand, in addition to high extraction efficiencies, the short extraction times used for MAE of approximately 30 min and 50 min for UAE are also suitable for green chemistry by providing significant energy savings compared to the approximately 8 h spent for SAE. This also prevents decomposition caused by exposure to long‐term heat treatment. Although the results obtained with the MAE and UAE systems were comparable, the antioxidant activity and curcuminoid content of the extract obtained with the UAE system were higher. Likewise, Wakte et al. (2011) showed that using EtOH as a soaking solvent, UAE provided a higher extraction rate constant and total yield than MAE in the extraction of curcumin from *Curcuma longa* . Consistent with the results obtained in this study, Sravanya et al. (2023) found that the ultrasound‐assisted extraction process was more efficient than the microwave boiling process in the extraction of curcumin from turmeric. In another study, Li et al. (2014) optimized pulsed ultrasound and microwave‐assisted extraction processes for the extraction of curcuminoids. According to the results of the study, the yields of the pulsed ultrasound and microwave‐assisted extraction processes were close to each other, and the extraction efficiency with the pulsed ultrasound process was found to be slightly higher. In the continuation of the study, red lentil soup enriched with the addition of turmeric extract obtained by the UAE system was prepared, and the effects of turmeric addition and spontaneous protein–curcumin interaction were investigated.

### 3.5 Total Phenolic Content and Total Antioxidant Capacity of Enriched Red Lentil Soup Model Samples With Turmeric Extract

In this study, enriched red lentil soups were prepared as a base model food system with basic ingredients by adding turmeric extract at different ratios to observe the effects of curcumin addition to a protein‐rich food matrix and spontaneous protein –curcumin interaction on the final characteristics of model soup samples. The effects of turmeric extract addition to red lentil soups on the final total phenolic content before and after in vitro gastrointestinal digestion were investigated, and the results of the TPC analysis are presented in Table 7.

**TABLE 7:** Total Phenolic Contents (TPC) of red lentil soup samples.

Sample code	TPC (mg GAE/100 g FW)		
	Initial	After the in vitro digestion process	
		Gastric phase	Intestinal phase
R.L + W	10.2 ± 1.3^b^	40.3 ± 2.3^ab^	205.1 ± 17.4^ab^
R.L + W + O.O	16.3 ± 6.1^b^	34.3 ± 5.1^ab^	177.7 ± 4.1^bc^
R.L + W + 5% T.E	19.6 ± 0.1^b^	43.4 ± 8.6^a^	158.0 ± 1.9^c^
R.L + W + 10% T.E	19.6 ± 0.9^b^	39.6 ± 1.3^ab^	155.0 ± 52.2^c^
R.L + W + O.O + 5% T.E	59.4 ± 7.5^a^	23.9 ± 0.4^b^	168.1 ± 9.3^bc^
R.L + W + O.O + 10% T.E	49.6 ± 1.8^a^	38.2 ± 1.7^ab^	226.4 ± 3.4^a^

*Note:* Data represent average quantities ± standard deviation. Different letters in the columns represent statistically significant differences (*p* < 0.05).

The addition of turmeric extract to red lentil soups prepared with only red lentils and water resulted in a considerable improvement (~2‐fold) in the total phenolic content of the final products before in vitro gastrointestinal digestion compared to (R.L + W) control sample. Similarly, higher TPC and total flavonoid content were obtained in lentil soups with the addition of turmeric powder in the study of Rozan et al. (2018). However, in this study, the addition of different amounts of turmeric extract had no effect on the final total phenolic content of the (R.L + W + 5% T.E) and (R.L + W + 10% T.E) samples and provided the same values as 19.6 mg GAE/100 g fresh weight (FW) in the initial samples. In the study by Liu et al. (2023), extracellular vesicles in various fruit and vegetable juices were loaded with curcumin, and relatively high encapsulation efficiencies were obtained for all samples at lower curcumin concentrations. However, a decrease in encapsulation efficiency was observed when the curcumin concentration was increased, especially above a certain critical value. This result was explained by the fact that the colloidal particles in the juices became saturated with curcumin and were therefore unable to solubilize any more curcumin. Therefore, the results obtained in this study can be explained by the same amount of protein source found in both (R.L + W + 5% T.E) and (R.L + W + 10% T.E) samples. Although a higher amount of turmeric extract was found in (R.L + W + 10% T.E) sample, the potential complexes that could be formed between curcumin and protein were at the same level in both samples.

When compared to the TPC analysis results after the in vitro digestion process, the (R.L + W + 5% T.E) sample had a higher TPC value compared to that of (R.L + W + 10% T.E) sample in both gastric and intestinal phases. In addition, a generally higher level of TPC was determined in the (R.L + W) control sample than in the fortified samples after the in vitro digestion process. These results may be due to the low solubility and higher degradation of curcumin during digestion, which leads to an obstacle in the formation of complexes with proteins, and insufficient digestion of protein compounds resulting in limited release of curcumin at the end of the in vitro digestion process. It is also known that curcumin is a heat‐sensitive compound and the degradation rate of curcumin is accelerated when the temperature increases (Tang 2020). Thus, this may also be due to the fact that curcumin, which does not form complexes during the heat treatment applied to prepare the soup samples, is more easily degraded at high temperatures than protein‐bound curcumin.

Furthermore, to investigate the effect of the presence of oil in the prepared samples due to the lipophilic nature of curcumin, another sample group was prepared with the addition of olive oil. A higher TPC value was detected in the (R.L + W + O.O) control sample compared to the (R.L + W) control sample in the initial and intestinal phases. It is well established that olive oil contains a high concentration of phenolic compounds, particularly oleuropein, hydroxytyrosol, and tyrosol, which were shown to possess significant antioxidant and radical scavenger properties (Tuck and Hayball 2002; Servili et al. 2009; Tripoli et al. 2005). Consequently, the elevated TPC values similarly observed in the results of antioxidant analysis in soup samples containing olive oil can be attributed to the fact that olive oil is a substantial source of phenolic substances when contrasted with samples prepared without olive oil. Moreover, in the initial and intestinal phases, (R.L + W + O.O + 5% T.E) and (R.L + W + O.O + 10% T.E) samples generally had a higher level of TPC than (R.L + W + 5% T.E) and (R.L + W + 10% T.E) samples and also their control sample (R.L + W + O.O). This result can be attributed to the potential ability of olive oil to provide solubility and stability of curcuminoids, which serves as a medium for the incorporation of turmeric extracts, attributable to their lipophilic nature. Additionally, the incorporation of olive oil into emulsions for lipophilic bioactive compounds, as evidenced in the studies conducted by Nemli et al. (2023) and Li et al. (2016), resulted in an increase in the total phenolic content and antioxidant activity. The underlying mechanism for these observations has been attributed to the inherent antioxidant phenolic compounds present in olive oil, which have the capacity to reduce and/or inhibit the oxidation of other substances within the gastrointestinal tract. When examining the results obtained in the gastric phase, a lower total phenolic content was detected in (R.L + W + O.O + 5% T.E) and (R.L + W + O.O + 10% T.E) samples than in (R.L + W + 5% T.E) and (R.L + W + 10% T.E) samples and also in the (R.L + W + O.O) sample. The obtained results may be due to an incomplete or partial digestion of olive oil in the gastric phase. It is also expected that curcumin would be adsorbed at the oil–water interface (the surface of the oil droplet) rather than within the oil phase, as the aliphatic chain structure of curcumin imparts hydrophobic characteristics to the molecule. Moreover, it has been shown that the stability of curcumin encapsulated in conventional oil‐in‐water emulsions can decrease with increasing temperature, particularly at neutral and alkaline pH (Kharat et al. 2017; Sabet et al. 2021). Therefore, although the TPC values of enriched soup samples with olive oil were higher than those of enriched soup samples without olive oil, they were found to be lower than expected. This may arise from the fact that the absorption and stability of curcumin were lower than envisaged due to the heat treatment applied during the preparation of soup samples and the addition of bulk oil instead of an emulsion form to the samples, resulting in a smaller absorption area for curcumin.

A comparison of the TPC values obtained from the in vitro digestion phases showed that the intestinal phase had higher TPC values than the gastric phase for all samples. The differences between the TPC values observed in the gastric and intestinal phases showed a range of differences, with a ratio of 3.6 to 7.0‐fold. Similarly, in the study of Günal‐Köroğlu et al. (2022), interactions between lentil protein and onion skin phenolics were investigated, and lower TPC values were found in the gastric phase compared to the intestinal phase. The lower TPC values observed in the gastric phase can be attributed to the low solubility of polyphenol‐protein complexes, which occurred at pH values of 0.3–3.1 units below the isoelectric point of the proteins. This is due to the fact that lower pH levels may provide more binding sites on the protein without affecting the binding constant, hinting that the nature of the binding was probably not ionic (possibly of hydrophobic nature) at low pH (Ozdal et al. 2013). The observation that the TPC values were higher in the intestinal phase than in the gastric phase for all combinations of (R.L. + T.E) (Table 7) may be attributed to the fact that protein‐phenolics interactions assume a more ionic (non‐covalent) character at higher pH, naturally depending on the type of protein and phenolic compound. As regards the type of bonding of curcumin tested with various proteins, non‐covalent, hydrophobic, and hydrogen‐bonding interactions come to the front (Guan et al. 2021).

CUPRAC and DPPH analyses were also carried out to investigate the effect of turmeric extract fortification on the total antioxidant activity of the final samples, and the results are shown in Table 8. Based on the results of CUPRAC and DPPH analyses, (R.L + W + 5% T.E) and (R.L + W + 10% T.E) samples generally had a higher total antioxidant efficiency than (R.L + W) samples both before and after the in vitro digestion process. When (R.L + W + 5% T.E) and (R.L + W + 10% T.E) samples were compared, higher CUPRAC and DPPH results were obtained in the (R.L + W + 5% T.E) sample than in the (R.L + W + 10% T.E) sample for the initial phase in concordance with their results of TPC analysis. For olive oil‐containing fortified red lentil soup samples, similar trends with TPC results were observed in the soup sample group prepared without olive oil. However, some exceptions to these results in this study may be caused by the degradation of curcumin during heat application, incomplete formation of curcumin‐protein complexes, insufficient protein counterpart to form complexes with curcumin in soup samples containing a higher amount of curcumin, incomplete digestion of proteins and oils with digestive enzymes, possible interactions between product ingredients and digestive enzymes, and possible additional interactions with reagents used in analyses. It is known in the relevant literature that curcumin‐protein hydrophobic interactions occur as a result of conflicting motivations of enthalpic and entropic change; with an increase in temperature, the endothermic process may become favorable by an increase in entropy as a result of the release of water molecules from the solvation layer of free curcumin and protein (Lelis et al. 2020). In the case of a higher percentage of T.E., complex formation may be thermodynamically hindered due to the entrapment of water of hydration within the pores of the macromolecular aggregates; when this hydration water is not released, there remains less entropic motivation for a stable complex.

**TABLE 8:** Antioxidant capacity values for red lentil soup samples.

Sample code	Antioxidant capacity (mg TR/100 g FW)					
	CUPRAC			DPPH		
	Initial	After the in vitro digestion process		Initial	After the in vitro digestion process	
		Gastric phase	Intestinal phase		Gastric phase	Intestinal phase
R.L + W	18.0 ± 0.4^b^	9.9 ± 0.9^ab^	84.1 ± 6.7^b^	32.9 ± 0.3^c^	20.9 ± 0.8^a^	45.3 ± 0.2^c^
R.L + W + O.O	49.5 ± 1.3^b^	10.0 ± 2.8^ab^	172.5 ± 15.6^a^	35.1 ± 0.1^bc^	20.8 ± 0.4^a^	46.9 ± 0.3^abc^
R.L + W + 5% T.E	33.2 ± 1.1^b^	12.3 ± 0.4^ab^	86.4 ± 26.3^b^	39.6 ± 5.0^a^	21.8 ± 0.3^a^	45.2 ± 1.6^c^
R.L + W + 10% T.E	32.4 ± 0.9^b^	13.2 ± 0.9^a^	73.8 ± 12.2^b^	38.1 ± 1.1^ab^	20.9 ± 0.6^a^	49.3 ± 1.9^a^
R.L + W + O.O + 5% T.E	145.0 ± 35.2^a^	8.0 ± 0.4^b^	165.0 ± 63.8^a^	32.6 ± 0.9^c^	20.7 ± 1.3^a^	46.1 ± 2.3^bc^
R.L + W + O.O + 10% T.E	117.0 ± 11.0^a^	11.8 ± 0.0^ab^	196.8 ± 10.5^a^	34.7 ± 3.1^bc^	20.6 ± 1.5^a^	48.5 ± 0.1^ab^

*Note:* Data represent average quantities ± standard deviation. Different letters in the columns represent statistically significant differences (*p* < 0.05).

Overall, various studies in the literature have proven that encapsulation applications are a promising way to improve the bioaccessibility and bioavailability of curcumin, but more information is needed on the effects of the consumption of encapsulated compounds within the human body. Therefore, the direct addition of curcumin to the food matrix, especially rich in protein, may be a good option for designing functional foods with health‐promoting effects. However, further studies are required to better understand the effects of curcumin fortification in foods, their interactions with other food components, and thus their impact on the nutritional value and health aspects of the food to which it is added.

## 4 Conclusion

In this study, MAE and UAE processes for antioxidant and curcuminoid extraction from turmeric were optimized and modeled using RSM. For each process, the effects of four important operational factors on antioxidant and curcumin extraction processes were investigated, and the conditions were determined to obtain an extract with the most favorable characteristics. The antioxidant activities of the extracts obtained by the automated SAE method, as well as the MAE and UAE processes under optimized conditions for maximal antioxidant extraction, were determined spectroscopically, and their curcuminoid contents were determined chromatographically. The highest TAC and CC yields were obtained at a 200 μm particle size, 100°C temperature, 30 min time, and 20% water in ethanol conditions for MAE. The highest TAC and CC yields were obtained at a 200 μm particle size, 48 min time, *G* = 600 W ultrasonic power, and 26% water in ethanol conditions for UAE. The most effective process in terms of both antioxidant activity and curcuminoid content was chosen to be UAE. Finally, red lentil soup, as a model food, was enriched with extracts obtained by the UAE process, and the effects of curcumin addition to a protein‐rich food matrix and spontaneous protein‐curcumin interaction were investigated. Fortification of red lentil soup samples with turmeric extract resulted in approximately 2‐fold and 3–3.5‐fold higher TPC values in undigested fortified samples without and with olive oil, respectively. Similar improvements were also observed in the TPC values of the digested fortified samples and in the TAC values of fortified samples before and after in vitro digestion. It has been demonstrated that the direct addition of curcumin to the food matrix, especially one rich in protein, is a good option for designing functional foods with health‐promoting effects. It was revealed that the obtained extracts can be considered natural food additives and have great potential for the design of new functional food formulations with high antioxidant activity. This study demonstrates that the optimization of MAE and UAE processes is not only crucial for achieving high extraction yields but also serves as a foundation for designing processes aligned with the principles of green chemistry. Furthermore, the extracts obtained from these optimized processes have shown potential for application as natural food additives, with significant potential for the development of novel functional food formulations possessing high antioxidant activity.

## Author Contributions

**Musa Yaman:** formal analysis (equal), investigation (equal), methodology (equal). **Sude Nur Arslan:** formal analysis (equal), investigation (equal), methodology (equal). **Gözde Gençay:** formal analysis (equal), investigation (equal), methodology (equal). **Elifsu Nemli:** formal analysis (equal), investigation (equal), methodology (equal). **Müge Yermeydan Peker:** methodology (equal), resources (equal), validation (equal). **Furkan Burak Şen:** conceptualization (equal), investigation (equal), resources (equal). **Esra Capanoglu:** conceptualization (equal), investigation (equal), methodology (equal). **Mustafa Bener:** supervision (equal), writing – original draft (equal). **Reşat Apak:** supervision (equal), writing – review and editing (equal).

## Conflicts of Interest

The authors declare no conflicts of interest.

## Data Availability

Data available on request from the authors.
